# Early-Onset Osteoporosis

**DOI:** 10.1007/s00223-021-00885-6

**Published:** 2021-07-08

**Authors:** Outi Mäkitie, M. Carola Zillikens

**Affiliations:** 1 Children’s Hospital, Pediatric Research Center, University of Helsinki and Helsinki University Hospital, Helsinki, Finland; 2 Research Program for Clinical and Molecular Metabolism, Faculty of Medicine, University of Helsinki, Helsinki, Finland; 3 Folkhälsan Research Center, Biomedicum Helsinki, P.O. Box 63, FI-00014 Helsinki, Finland; 4 Department of Internal Medicine, Erasmus University Medical Center, 3015 Rotterdam, The Netherlands

**Keywords:** Fragility fractures, Osteogenesis imperfecta, Early-onset osteoporosis, Osteoporosis in children

## Abstract

Osteoporosis is a skeletal disorder with enhanced bone fragility, usually affecting the elderly. It is very rare in children and young adults and the definition is not only based on a low BMD (a *Z*-score < − 2.0 in growing children and a *Z*-score ≤ − 2.0 or a *T*-score ≤ − 2.5 in young adults) but also on the occurrence of fragility fractures and/or the existence of underlying chronic diseases or secondary factors such as use of glucocorticoids. In the absence of a known chronic disease, fragility fractures and low BMD should prompt extensive screening for secondary causes, which can be found in up to 90% of cases. When fragility fractures occur in childhood or young adulthood without an evident secondary cause, investigations should explore the possibility of an underlying monogenetic bone disease, where bone fragility is caused by a single variant in a gene that has a major role in the skeleton. Several monogenic forms relate to type I collagen, but other forms also exist. Loss-of-function variants in *LRP5* and *WNT1* may lead to early-onset osteoporosis. The X-chromosomal osteoporosis caused by *PLS3* gene mutations affects especially males. Another recently discovered form relates to disturbed sphingolipid metabolism due to *SGMS2* mutations, underscoring the complexity of molecular pathology in monogenic early-onset osteoporosis. Management of young patients consists of treatment of secondary factors, optimizing lifestyle factors including calcium and vitamin D and physical exercise. Treatment with bone-active medication should be discussed on a personalized basis, considering the severity of osteoporosis and underlying disease versus the absence of evidence on anti-fracture efficacy and potential harmful effects in pregnancy.

## Introduction

Osteoporosis is a skeletal disorder mainly affecting elderly people and characterized by low bone mass and abnormal bone microarchitecture, resulting in enhanced skeletal fragility and increased risk of fractures [1]. The fractures with ensuing morbidity and mortality have significant personal and economic implications worldwide. Osteoporosis was previously considered an illness affecting mainly post-menopausal women, but primary and secondary osteoporosis have more recently emerged also as important pediatric disorders [2, 3]. The prevalence of osteoporosis in young persons is considered to be low but the true prevalence is unknown and dependent on the applied definition.

A low areal BMD on a DXA scan without fractures or without underlying diseases may not necessarily imply increased bone fragility and is usually associated with a low risk of fractures in the short term [14]. This situation is different in patients with a chronic disease that impacts bone health, such as inflammatory diseases [rheumatoid arthritis (RA), inflammatory bowel disease (IBD), chronic obstructive pulmonary disease (COPD)] and diseases related to poor nutrition or nutritional deficiencies [celiac disease, cystic fibrosis (CF), anorexia nervosa (AN)] and endocrine disorders (Cushing’s syndrome, hyperparathyroidism, hyperthyroidism, type 1 diabetes, hypogonadism) [3–5]. These can lead to bone fragility due to the underlying disease, disease-associated co-morbidities, like malnutrition, or due to the applied therapies. Such secondary disturbances in modeling and remodeling of the skeleton during growth will have persisting long-term consequences later in life with reduced peak bone mass and structural deterioration of bone [6–8]. The prevalence of osteoporosis, defined as a BMD *Z*-score below − 2.0, has recently been reported to be as high as 45% in adults with Cushing’s disease [9] and in young adults with cystic fibrosis [10].

In young adults presenting with fragility fractures and low BMD without known chronic diseases, an underlying secondary factor can often be identified, depending on the depth of investigations and the type of hospital setting. However, fragility fractures may also be a presenting symptom of a monogenic form of osteoporosis. Fractures presenting in childhood or early adulthood without an evident secondary cause should prompt investigations for an underlying monogenetic bone disease such as osteogenesis imperfecta (OI) and several recently discovered other genetic entities. The diagnosis of idiopathic osteoporosis should thus be reserved only to cases where secondary and known monogenic causes have been appropriately excluded.

In this narrative review we discuss the definition of early-onset osteoporosis and its most common non-genetic and genetic causes and present a plan of action for evaluation and treatment.

## Definition of Early-Onset Osteoporosis

Skeletal mass increases rapidly during childhood and especially adolescence; 90% of peak bone mass is acquired by age 18 [8, 11, 12]. Peak bone mass has been regarded an important determinant of osteoporosis and fracture risk later in adulthood [11]. In adults, osteoporosis is defined as a BMD measured by dual energy X-ray absorptiometry (DXA) with a *T*-score below or equal to − 2.5. For persons younger than 50 years both *T*-scores and *Z*-scores are used. The International Society for Clinical Densitometry (ICSD) proposed a BMD Z-score below or equal to − 2.0 to define low BMD in those below 40 years [13]. The IOF also defines low BMD in persons below age 20 years as *Z*-scores below − 2.0 and as *T*-score of below − 2.5 in those 20 years and older in association with a chronic disease known to affect bone metabolism [14].

In children the diagnosis of osteoporosis is more complex and BMD measurement by DXA is significantly impacted by the patient’s height and timing of pubertal development. Fractures are also common in childhood, especially around the time of rapid growth prior and during puberty, and one needs to take into consideration the normal fracture pattern and prevalence before regarding fractures as a sign of osteoporosis. Therefore, the diagnosis is based not only on low BMD that has been appropriately adjusted for height and/or skeletal maturity but also requires a fracture history indicative of higher-than-normal bone fragility and knowledge on underlying diseases and secondary factors.

Table 1 shows the criteria for a DXA scan assessment and for the diagnosis of early-onset osteoporosis in children and adults. In children, recurrent fractures are often the result of deficient calcium intake or vitamin D deficiency [15] but also other acquired or genetic disorders with disturbed mineral homeostasis (e.g., hypophosphatemia, hyperparathyroidism, osteomalacia, hypophosphatasia) need to be considered [16, 17]. Importantly, several monogenic forms of early-onset osteoporosis exist, as will be discussed more in detail in this review. Pathological fractures may occur due to localized bone abnormalities such as Paget’s disease of bone, fibrous dysplasia or malignancy. These can be excluded by appropriate laboratory work-up and radiological examinations. Secondary causes of osteoporosis should be searched for, especially in young individuals with low BMD and fragility fractures and these investigations should contain a minimum set of laboratory investigations and be guided by the patient’s symptoms and previous medical history.

**Table 1:** Indication for performing DXA scan in children and young adults below age 50 and definition of osteoporosis

Indication for DXA scan
≥ 2 fragility fractures (after low to moderate energy trauma) before age 10 years
> 2 fragility fractures (after low to moderate energy trauma)
Fracture(s) at an unusual site (spine, hip)
> 2 fragility fractures with a family history of fractures
> 2 fragility fractures with extraskeletal signs of OI (blue sclerae, joint laxity)
A fragility fracture in patients with a chronic disease or use of glucocorticoids
Diagnosis of osteoporosis
Any of the above situations plus
BMD *Z*-score of the spine or total body < − 2.0 adjusted for height and pubertal status in growing children
BMD *Z*-score < − 2.0 or *T*-score < − 2.5 of the spine or femur neck when adult height has been reached

## Early-Onset Osteoporosis as a Sequelae of Other Illness

Many chronic diseases of childhood and young adulthood can lead to low BMD. These include inflammatory diseases such as RA, IBD, COPD and diseases related to poor nutrition or nutritional deficiencies (anorexia nervosa, celiac disease, cystic fibrosis, vitamin D deficiency), endocrine disorders (Cushing’s syndrome, hyperthyroidism, hyperparathyroidism, type 1 diabetes mellitus, hypogonadism) and chronic infectious (HIV), renal, liver or neurological diseases. Table 2 lists major causes of secondary osteoporosis in the young. In the following paragraphs we will briefly discuss some of these conditions potentially leading to early-onset osteoporosis, including inflammatory diseases (RA, IBD and COPD), cancer, and anorexia nervosa, as well as osteoporosis related to glucocorticoid therapy. In addition, we summarize some recent findings in pregnancy and lactation-associated osteoporosis (PLAO).

**Table 2:** Secondary factors and underlying diseases in early-onset osteoporosis

Endocrine diseases	Inflammatory/rheumatic diseases	Malnutrition or malabsorption	Medications
Acromegaly	COPD	Anorexia nervosa	Anticonvulsants
Cushing’s disease/syndrome	IBD	Calcium and Vitamin D deficiency	Antidepressants?
Diabetes mellitus (Type 1 and 2)	Rheumatoid arthritis	Celiac disease	Aromatase inhibitors with OST
Growth hormone deficiency	Sarcoidosis	GI surgery	Chemotherapy
Hyperprolactinaemia	Systemic lupus erythematosus	IBD	Fall-related medication e.g., sedatives
Hypogonadism			HAART
Delayed or absent puberty	Organ failure/transplantation	Metabolic diseases	Elagolix
Hyperparathyroidism	Bone marrow transplantation	Gaucher's disease	Glucocorticoids
Hyperthyroidism	Cystic Fibrosis	Glycogen storage disease	H2-receptor inhibitors
Hypopituitarism	Chronic liver disease	Hypophosphatasia	Heparin
	Chronic kidney disease	Hypophosphataemia	LHRH agonists
	Hematologic diseases	Homocystinuria	Medroxyprogesterone acetate
Diverse	Hemochromatosis	Mucopolysaccharidoses	Protonpump inhibitors
Alcohol abuse	Multiple Myeloma	Pompe disease	Tamoxifen
Calcium and Vitamin D deficiency	Haemophilia		Thiazolidinediones
Cancer	Leukemia		Thyroid hormone excess
Cerebral Palsy	Lymphoma		
Fall-related diseases and medications	MGUS		
Female Athlete Triad	Thalassemia Major		
HIV	Mastocytosis		
Immobility	Solid organ transplantation		
DMD and other myopathies			
Pregnancy and breastfeeding			

*COPD* chronic obstructive pulmonary disease, *DMD* Duchenne muscular dystrophy, *HAART* highly active antiretroviral therapy, *HIV* human immunodeficiency virus, *IBD* inflammatory Bowel Disease, *MGUS* Monoclonal gammopathy of unknown significance, *OST* Ovarian suppression therapy, *PPI* proton pump inhibitors

### Osteoporosis in Chronic Inflammatory Diseases

The etiology of fragility fractures and low BMD in chronic inflammatory diseases rheumatic disorders, chronic lung diseases, inflammatory bowel disease (Crohn’s disease, Ulcerative Colitis) is also multifactorial, including effects of the underlying disease, systemic inflammation, use of glucocorticoids, low body weight, malabsorption, low physical activity and delayed puberty and/or secondary amenorrhea. At a young age, the disease will lead to a decrease in peak bone mass while at older age there may be increase bone loss [18, 19]. The most important treatment is that of the underlying disease and supplementation of calcium and vitamin D. There are very few RCTs with osteoporosis medication on fracture outcome. A meta-analysis in inflammatory bowel disease including 13 RCTs en 925 men and women, of which only 10% was pre-menopausal, showed an increase in BMD and a decrease in fractures with bisphosphonates [20].

### Osteoporosis in (Breast) Cancer

Breast cancer is the most frequent cancer in women and early diagnosis and improved treatment has resulted in recent years in increased survival with more side effects from cancer treatments including bone loss and fractures. Mechanisms for bone loss include hypogonadism through chemotherapy, the direct toxic effects of chemotherapy itself, endocrine therapy (gonadotropin releasing hormone analogs, aromatase inhibitors and tamoxifen) as well as the general effects of being ill, loss of body weight and decreased physical activity [21]. Treatment with tamoxifen prevents bone loss in postmenopausal women but is deleterious for bone health in premenopausal women, resulting in a 75% increased fracture risk [22]. In all pre- and postmenopausal women initiating aromatase inhibitor treatment and in premenopausal women initiating tamoxifen, fracture risk should be assessed and recommendations regarding exercise and calcium and vitamin D supplementation should be given. Guidelines for indications of starting bone-active medication have been published, mostly based on expert opinion and there are no clinical trials on fracture prevention in premenopausal women [21, 23].

### Osteoporosis in Anorexia Nervosa (AN) and the Female Athlete Triad

Anorexia Nervosa (AN) is serious eating disorder affecting 0.3–3% of girls and young women, but also boys and men can be affected. Patients with AN have a reduced BMD and an increased risk of osteoporosis (up to 40%) and about 30% has a history of prevalent fractures [24, 25]. Bone loss results from low body fat mass and a decreased energy intake resulting in hypothalamic amenorrhea and hypogonadism with complex neuroendocrine hormone dysregulation (such as increases in ghrelin, cortisol, PYY and growth hormone (GH) with GH resistance, and decreases in levels of insulin, IGF1 and oxytocin) and changes in levels of adipokines (low leptin and high adiponectin levels) [26]. There may also be a potential negative influence on bone of increased bone marrow adiposity and preferential development of mesenchymal stem cells towards adipocytes instead of osteoblasts. AN has the highest impact on BMD at a young age when the insult to the bone happens during formation of peak bone mass. The prevalence of low BMD and increased risk of fractures is determined by age at diagnosis and menarche, duration of amenorrhea and BMI. Weight gain with regain of menses is the most important treatment goal for BMD gain but deficits often persist [26]. Other potential treatment options include (transdermal) estrogens, bisphosphonates and teriparatide, that have shown to increase BMD in small clinical trials, but no data on fracture prevention are available [27].

Young athletes who participate in intense athletic activities like running and ballet may have reduced energy intake, amenorrhea, and low BMD, collectively called the ‘Female Athlete Triad’. When these young females are not considered to have AN, this triad is often not recognized as a cause for fractures and low BMD. Female athletes often present with one or more of these triad components, and early intervention is essential. In a consensus statement from 2014 a set of recommendations was presented to provide clinical guidelines for screening, diagnosis, and treatment of the Female Athlete Triad [28].

### Glucocorticoid-Induced Osteoporosis

Glucocorticoid-induced osteoporosis in children and young adults is usually seen in patients with immune-mediated diseases, such as rheumatic disorders, chronic lung diseases, inflammatory bowel disease (Crohn’s disease, ulcerative colitis), chronic liver and kidney diseases, skin diseases, and in organ transplantation, diseases that are in themselves also a cause of osteoporosis. The negative effects of the glucocorticoids on bone are multifactorial, including increased apoptosis of osteoblasts and osteocytes with decreased apoptosis of osteoclasts, negative effects on muscle function, sex-steroids and a decreased calcium absorption in the gut and decreased calcium re-absorption in the kidney. Despite the negative effects on bone health, they may also have some favorable effects on bone by controlling the activity of the underlying disease [29] although this has not been adequately studied in young adults and children. A clinically significant number of children with rheumatic disorders developed incident vertebral fractures in the 3 years after starting glucocorticoids (incidence rate 4.4 per 100 person-years) [30]. Almost half of the fractures were asymptomatic and thus would not have come to clinical attention in the absence of radiographic screening. Guidelines from the American College of Rheumatology in 2017 advise to perform clinical fracture risk assessment in all children and young adults within six months of starting glucocorticoid therapy and to perform a DXA scan in young adults below 40 years of age (but not in children) when there is a history of osteoporotic fractures or other significant risk factors for fracture [29]. In adults of 40 years or above they advise to use FRAX with glucocorticoid dose correction and BMD testing within six months of starting glucocorticoids [31].

### Pregnancy and Lactation

During a normal pregnancy there is a temporary decrease in BMD with a stronger loss during breastfeeding. In the spine the loss of BMD is about 5–10% with a spontaneous recovery within 6–12 months. Several case reports have been published on the occurrence of “transient osteoporosis of the hip” (TOH) and of vertebral fractures during pregnancy and lactation (pregnancy and lactation-associated osteoporosis, PLAO). PLAO is a severe type of premenopausal osteoporosis which predominantly occurs in the last trimester of pregnancy or immediately postpartum. Almost 25% of patients with PLAO will sustain a subsequent fracture, and this fracture risk correlates with the number of fractures at the time of diagnosis [32]. Sometimes underlying secondary factors can be found. There is often a spontaneous improvement in BMD after delivery and cessation of breast feeding. Recent studies using bone biopsies suggest a possible defect in the functioning osteoblasts [33]. Pre-existing secondary causes of osteoporosis should always be ruled out and while some patients will improve spontaneously, others will need treatment with either antiresorptives or with anabolic treatment [19]. In some patients an underlying genetic predisposition may be identified, e.g., with pathogenic variants in *LRP5*, suggesting a pre-existing monogenetic form of osteoporosis with an exacerbation due to pregnancy, resulting in vertebral fractures [34]. PLAO may thus present as a rare presentation of early-onset osteoporosis that becomes apparent during the times of skeletal stress of pregnancy and lactation. After exclusion of secondary and genetic causes a persisting low BMD more than 6 months after cessation of pregnancy and lactation may indicate idiopathic osteoporosis.

## Idiopathic Osteoporosis

This is a diagnosis per exclusionem when no underlying chronic disease or secondary factors can be found for fragility fractures associating with a low BMD. This condition is most likely multifactorial and should be differentiated from situations in which low BMD is present without fractures e.g., in constitutionally lean persons. Bone biopsies may show decreased bone formation in some patients [35]. Using high-resolution pQCT some similarities were found consistent with mild forms of OI with a reduction in volumetric BMD and changes in microstructure, however without changes in bone geometry [36]. Vertebral fractures are common in idiopathic male osteoporosis and have been associated with increased cortical porosity in iliac crest bone biopsies [35]. It is likely that a proportion of patients with idiopathic osteoporosis may have an underlying genetic cause.

It is important to bear in mind that in several instances so-called idiopathic osteoporosis has in fact a monogenic cause that can escape detection when only limited genetic testing is performed. Using a NGS panel in 123 young adults, rare or novel variants were found in 11 patients in the included candidate genes (*COL1A1*, *WNT1*, *PLS3* and *DKK1*) as well as a high prevalence of known pathogenic variants in *LRP5* in 22 patients [37]. Variants in *LRP5* have previously been identified in children [38] and in adult males with idiopathic osteoporosis [18]. The diagnosis of idiopathic osteoporosis should thus be reserved only to cases where secondary and known monogenic causes have been appropriately excluded.

## Genetic Determinants of Osteoporosis

During childhood, the skeleton undergoes rapid changes in both longitudinal growth and in bone modeling. Renewal of the bone tissue (remodeling) continues even after growth plates have fused and adult height has been reached. The remodeling process requires coordinated activity of osteoblasts, osteoclasts and osteocytes and integrity of the various signaling pathways that control the differentiation and function of these bone cells. In addition, adequate supply of minerals and normal hormonal control of mineral homeostasis are needed for appropriate bone mineralization [39].

Because of the complexity of the cellular networks in the skeleton, the genetic defects leading to skeletal fragility are numerous and variable in presentation. Monogenic low bone mass disorders can result e.g., from defects in osteoblastic bone formation, from increased osteoclastic bone resorption, or from abnormalities in the mineralization process. Osteogenesis Imperfecta (OI), although being a rare disease, is the most common inherited bone disease with low bone mass and increased fractures, often associated with some extra-skeletal features such as blue sclerae [40]. OI is caused by defects in type I collagen itself, or its posttranslational modification. In more than 90% of OI cases the gene defect involves one of the two genes (*COL1A1* or *COL1A2*) encoding the two α-chains of type I collagen while the remaining cases show a very heterogeneous genetic background and various inheritance patterns [41, 42]. In the present article we have chosen to focus on other types of monogenic bone fragility that are not directly caused by defects in type I collagen and should be considered in differential diagnosis when evaluating a child or a young adult with early-onset osteoporosis.

## Monogenic Bone Fragility Due to Impaired WNT-Signaling Activity

Genetic entities with defective WNT signaling have emerged as an important subgroup of monogenic bone fragility disorders. The spectrum of monogenic skeletal disorders directly related to the WNT signaling pathway is still increasing and includes disorders with both high and low bone mass [43]. Some genetic forms lead to severe skeletal fragility, impaired growth and deformities already in childhood and genetic evaluations are usually initiated in infancy or early childhood. However, others present only in adolescence or early adulthood with increased susceptibility to fractures without any significant extra-skeletal manifestations, height deficit, deformities, or laboratory abnormalities. For example, patients with biallelic *LRP5* and *WNT1* mutations present with severe skeletal fragility, growth impairment and deformities in early childhood [44, 45], while subjects with heterozygous mutations in these genes often have normal growth, lack deformities but sustain fractures and have low BMD during later childhood or in early adulthood [45–47].

### *LRP5*

Biallelic rare variants in *LRP5* cause the autosomal recessive osteoporosis–pseudoglioma syndrome (OPPG, MIM 259770), characterized by generalized childhood-onset osteoporosis and blindness [44]. Already in early studies on *LRP5* and OPPG, and in many studies thereafter, it has been noticed that carriers of heterozygous rare *LRP5* variants also have reduced bone mass but usually lack eye manifestations or may have a milder eye phenotype in the form of vitreoretinopathy [34, 44, 48]. Several studies have identified individuals with childhood or early adulthood onset symptomatic osteoporosis caused by rare heterozygous *LRP5* variants [38, 47]. In addition, even common *LRP5* single nucleotide variants have been linked to childhood bone mass accrual, childhood fractures, and peak bone mass in cohort studies and in genome-wide association studies on BMD and fractures [38, 49, 50].

Studies evaluating characteristics of autosomal dominant osteoporosis caused by rare heterozygous loss-of-function *LRP5* variants usually include only a small number of affected individuals. A recent study on a large cohort (372 individuals) of subjects with early-onset osteoporosis identified rare *LRP5* or *LRP6* variants in 8.3% [47]. Detailed assessment of skeletal characteristics and treatment responses in those harboring a rare variant showed significant heterogeneity both in bone parameters and in efficacy of therapies. Analysis of bone metabolism revealed low bone formation markers in individuals carrying rare *LRP5* or *LRP6* variants, in line with decreased WNT signaling [47]. Another study evaluating the impact of two common *LRP5* single nucleotide polymorphisms (rs4988300 and rs634008) on bone turnover markers in a cohort of 328 unrelated osteoporosis patients with or without fractures, found that the bone formation marker PINP levels and BMD were lower in patients with the GG genotype of rs4988300 and the TT genotype of rs634008 than in patients with the other genotypes [51]. However, no significant difference in b-CTX levels was observed between different genotypes.

In patients with osteoporosis-pseudoglioma syndrome due to biallelic *LRP5* variants the response to bisphosphonate treatment is usually good [52, 53], but little is known about the treatment responses in those harboring heterozygous *LRP5* variants. Studies have also explored whether common gene variants in *LRP5* could affect response to bisphosphonate treatment, but although some variants associated with baseline BMD, no effect on treatment response was observed [54, 55]. Analysis of osteoanabolic treatment with teriparatide in two individuals with an *LRP5* or *LRP6* variant indicated acceleration of bone turnover during treatment [47].

Studies have suggested that *LRP5* variants may also lead to altered insulin sensitivity, impaired glucose tolerance and hyperlipidemia [56–58] but the clinical relevance of this connection remains uncertain.

Functional studies have in some instances confirmed reduced WNT signaling by these *LRP5* variants [59]. However, as rare variants in several genes, including *LRP5*, can be found in the general population that are predicted to be (likely) pathogenic or of undetermined significance it is not always easy to link these variants to the patient’s osteoporosis, especially when segregation analysis in the family is not possible due to lack of large pedigrees or when family members have low BMD due to other (non-genetic) causes [47]. Rare pathogenic *LRP5* variants have also been described in patients with pregnancy- and lactation-associated osteoporosis (PLAO) [34].

### *WNT1*

Several WNT ligands are expressed in bone tissue and regulate bone homeostasis and are hence relevant for osteoporosis pathogenesis [60]. Based on genome-wide association studies on BMD and fractures, *WNT16* was discovered as an important ligand for WNT signaling in bone [61]. *WNT16* variants associate with cortical bone thickness, BMD, and osteoporotic fracture risk and may also impact peak bone mass [62–64]. However, *WNT16* variants have not been linked to monogenic osteoporosis, possibly implying that other WNT ligands have overlapping functions.

In 2013, we and several other groups identified *WNT1* as a key ligand to the WNT pathway in the regulation of bone formation and bone homeostasis. While biallelic loss-of-function mutations led to severe autosomal recessive OI-like phenotype with severe short stature, fractures, deformities and in some instances, developmental defects in the central nervous system, heterozygous *WNT1* mutations were reported to cause autosomal dominant osteoporosis [45, 65, 66]. Since then, several cases with recessive OI caused by *WNT1* mutations have been reported [67–70], confirming the severe OI type III -like phenotype in these patients. Ptosis has been suggested as a specific hallmark of this disease [67].

In contrast, *WNT1*-associated autosomal dominant early-onset osteoporosis, caused by heterozygous variants, has been reported less frequently. In our analyses involving a large Finnish cohort of 25 *WNT1* mutation-positive children and adults, all with the same heterozygous missense *WNT1* variant p.Cys218Gly, we have obtained in-depth information regarding the presenting features and progression of bone fragility, tissue-level bone characteristics, biomarkers and extra-skeletal manifestations. Affected children present in childhood usually with normal growth, only mildly reduced BMD but often with frequent long bone fractures and mild radiographic changes in long bone morphology, the fibulae being particularly thin [46]. Vertebral compression fractures are rare in childhood and early adulthood but practically all individuals have increased kyphosis and spinal compression fractures after the age of 50 years [71].

Histomorphometric analyses of transiliac bone biopsies demonstrated low-turnover osteoporosis [45]. Immunohistochemistry of bone biopsies showed altered expression of FGF23, sclerostin and phosphor-β-catenin and histology showed abnormal osteocyte morphology [72]. Quantitative back-scattering electron imaging (qBEI) showed heterogeneous matrix mineralization in children but homogeneous and increasing mineralization in adults [73]. Teriparatide treatment had only a minor effect on mineralization and seemed to increase bone marrow adiposity [73, 74]. Another study reported myelofibrosis in one young adult with *WNT1* osteoporosis and increased bone marrow fibrosis in other individuals with the same heterozygous *WNT1* variant [75]. It is unclear whether this is indicative of the importance of intact *WNT1* signaling for the bone marrow niche or a sign of imbalanced maturation of the hematopoietic stem cell—osteoblast lineage.

While traditional biomarkers for bone turnover tend to be normal in *WNT1* mutation-positive subjects with osteoporosis, the patients have a unique miRNA profile in serum [76]. In search for other potential biomarkers for this type of osteoporosis we showed that both intact and C-terminal FGF23 were significantly elevated in *WNT1* mutation-positive subjects, while concentrations of the two WNT pathway-associated markers Sclerostin and *DKK1* did not differ from age-matched controls [76].

A two-year teriparatide treatment in three adults showed increased bone formation but, as mentioned earlier, also a tendency to increased bone marrow adiposity [74]. Overall, the treatment results with conventional osteoporosis medications have not been optimal as several of the *WNT1* mutation-positive adults have developed significant skeletal pathology with extensive spinal compression fractures despite several years of treatment [71]. Novel anabolic treatments, targeting specifically the WNT pathway may provide improved treatment results. Evidence for this in humans is still lacking but experiments in the *WNT1* murine model, “the Swaying mouse” [77], suggest that sclerostin antibody is effective in increasing bone mass and decreasing peripheral fractures [78].

Although no major extra-skeletal features are seen in patients with heterozygous *WNT1* variants, there are features suggesting cartilaginous alterations, such as vertebral endplate deterioration with frequent Schmorl nodes and changes at the knee articular cartilage [71, 79]. In addition, bone marrow biopsies indicated increased reticulin and altered granulopoiesis as signs of abnormal bone marrow function [75].

## X-Chromosomal Osteoporosis Due to *PLS3* Mutations

In 2013 Dijk et al. described a novel monogenic form of osteoporosis that involved predominantly boys and men in five families [80]. The causative gene defect involved the *PLS3* gene, encoding Plastin 3. The gene’s X-chromosomal location explained why *PLS3* mutations affected mainly hemizygous males while the heterozygous females did not present significant bone fragility. Since the original description, several other families and single patients have been reported [81–89]. Based on these, it is evident, that *PLS3* mutations cause in affected males severe, early-onset and progressive osteoporosis predominated by multiple spinal compression fractures. Peripheral fractures are also common and may also present as atypical femur fractures after use of bisphosphonates [90, 91]. Despite being an X-chromosomal disorder, even females with a heterozygous *PLS3* variant can present with significant peripheral fractures and vertebral compressions especially later in adulthood [92]. However, Kämpe et al. [82] also described a young girl who presented with recurrent peripheral fractures, extremely low BMD (lumbar spine BMD *Z*-score − 6.6 at 6 years) and a heterozygous de novo *PLS3* variant. This indicates that *PLS3* variants should be considered especially in males but even in females with early-onset osteoporosis. Regarding the nature of the reported variants, the studies have identified both missense and nonsense variants but also partial or total deletions of the gene [83, 89] as well as a partial duplication of the gene [85] in individuals with early-onset osteoporosis.

The mechanisms leading from pathogenic *PLS3* variants to the clinical phenotype of the disorder have not yet been fully uncovered. *PLS3* plays an important role in the maintenance of the intracellular actin cytoskeleton. *PLS3* has been suggested to be important for the osteocytes’ mechanosensing properties. Osteocyte shape is dependent on actin filaments and osteocyte processes are rich in actin [93], suggesting that this actin-bundling protein could indeed be especially important for osteocyte function. *PLS3* has also been implicated in bone matrix mineralization. Matrix vesicles, crucial in the mineralization process, are formed by budding from the tip of mineralizing cell microvilli. These microvilli contain a dense bundle of cross-linked actin microfilaments as a structural core. Thouverey et al. showed that in a mineralizing osteocyte-like cell line *PLS3* was expressed both in the budding matrix vesicles and in the apical microvilli from which the vesicles were formed [94].

Detailed evaluation of transiliac bone biopsies obtained from individuals with *PLS3*-related osteoporosis show low bone turnover often with increased unmineralized osteoid [81, 82] and in quantitative backscattering electron imaging, a very variable mineralization pattern in childhood and more uniform increase in mineralization with age in adults [73, 83]. Evaluations of circulating biomarker profiles in patients with *PLS3*-related osteoporosis showed surprisingly normal conventional bone marker concentrations, increased *DKK1* concentration and a specific miRNA profile with alterations also in some miRNAs linked to the WNT signaling pathway and TGF-beta signaling pathway [76, 95].

Recently some data emerging from studies on a *PLS3* knock-out mouse model were described [96]. Based on μCT scanning, the *PLS3*-deficient mice exhibited moderate osteopenia at 12 weeks. More detailed skeletal evaluation at various ages revealed that *PLS3*-deficiency in mice only recapitulated the cortical bone phenotype of the human *PLS3*-related osteoporosis by negatively affecting the early stage of cortical bone acquisition, the cortical thickness in both tibia and femur being significantly reduced in *PLS3*-deficient mice in all age groups. In contrast, no significant differences between wildtype and *PLS3*-deficient littermates were detected in trabecular bone mass or in histomorphometric parameters at 12 weeks [96]. It therefore remains uncertain whether this model is suitable for e.g., preclinical trials testing various treatment options.

## Osteoporosis Caused by *SGMS2* Variants

The combination of early-onset osteoporosis and doughnut-shaped sclerotic skull lesions was described in 1974 [97]. This and subsequent reports confirmed the existence of a specific autosomal dominant disorder which was later termed as “osteoporosis with calvarial doughnut lesions” (CDL) (OMIM #126550) and was characterized by osteopenia, multiple pathologic fractures, elevated serum alkaline phosphatase, doughnut-shape calvarial lesions and dental caries. Mutations in the gene *SGMS2* were recently identified as the cause of this rare autosomal dominant disorder [98]. *SGMS2* encodes sphingomyelin synthase 2, an enzyme involved in sphingolipid metabolism. Sphingomyelin is a major lipid of the plasma membrane and enriched in microdomains of the plasma membrane that are critical for signal transduction. Mutations in *SGMS2* lead to changes in the sphingomyelin synthase enzyme function and, through mostly unknown mechanisms, to a significant disturbance in bone metabolism and mineralization.

Individuals with a heterozygous *SGMS2* mutation had sustained since childhood peripheral and spinal fractures [98]. Histomorphometric evaluation of patients’ bone biopsies showed a decrease in bone volume, reduced mineral content, heterogeneity of matrix mineralization, and importantly, a very disturbed matrix lamellarity with woven-bone appearance. Several subjects displayed neurological symptoms, transient facial nerve palsy being particularly common, suggesting that these extra-skeletal disease manifestations may be a distinctive feature of *SGMS2*-related osteoporosis [98].

Importantly, the phenotype varied significantly depending on the nature and location of the *SGMS2* mutation. The recurrent Arg50* stop-gain variant was reported in four unrelated families in the original publication [98]. It has thereafter been reported in at least two additional families with CDL [99] confirming this to be the “hot-spot” for osteoporosis-causing variants. In contrast, in two families a missense mutation in the same gene led to a much more severe disorder with spondylometaphyseal skeletal dysplasia, significant calvarial hyperostosis, severe short stature and skeletal fragility since early infancy [98]. Since only a few reports on mutation-positive subjects have been published, the full spectrum of *SGMS2* mutation-associated skeletal pathology remains to be elucidated.

## Early-Onset Osteoporosis as a Polygenic Disorder

Polygenic risk scores are used to take into consideration the sum effect of several gene variants that may contribute to the overall risk for a certain phenotype. A recent study on individuals with a significant childhood fracture history but no identifiable monogenic cause had an increased burden of common fracture risk alleles compared to the general population [100]. This suggests that some patients with presumed monogenic osteoporosis do in fact have a polygenic etiology for bone fragility, making diagnostics and genetic counseling much more demanding. Further research is required to develop clinically usable tools for estimating polygenic contribution to early-onset osteoporosis in an individual patient and family and to separate monogenic forms from polygenic forms. Interestingly, some attempts to identify the underlying genetic cause for osteoporosis in patients with presumed monogenic form of osteoporosis have in fact identified two or several rare and potentially pathogenic variants in the sequenced candidate genes, e.g., heterozygous variants in both *WNT1* and *PLS3* in the same individual [101]. These studies are limited by lack of functional data exploring the significance of the identified variants and the links between gene variants and the phenotype thus remain uncertain. With increasing genetic testing in various patient cohorts with mild to moderate early-onset osteoporosis it has become evident that more tools are needed also in clinical settings to determine the true significance of the detected rare variants.

## Evaluation of Osteoporosis

Once a diagnosis of osteoporosis has been established, based on the criteria suggested in Table 1, a thorough evaluation needs to take place. This involves a complete medical history and physical and laboratory examination to search for underlying chronic diseases and secondary factors. As discussed previously, many chronic diseases of childhood and young adulthood can lead to low BMD and fractures. These conditions often influence bone health and BMD through multiple mechanisms, including systemic inflammation, malnutrition, sex hormone deficiency, delayed puberty, and low mobility. Furthermore, several medications that are directly or indirectly harmful to bone, may be used, including glucocorticoids, anti-epileptic drugs or cancer treatment.

When no chronic condition is known, a thorough laboratory evaluation should be aimed at identifying secondary causes. The suggested laboratory tests for basic and extended screening are shown in Table 3. The main goal of this evaluation is to identify potential treatable secondary conditions. When no evidence of secondary factors can be found, a monogenetic cause should be considered and evaluated with appropriate genetic tools, as discussed in a separate paragraph. Bone turnover markers may help in diagnostic evaluation but are more helpful especially in adults in monitoring disease course and treatment response. Careful histological and histomorphometric evaluation of a transiliac bone biopsy is a standardized method to obtain detailed information regarding the bone metabolic activity and is often a very useful tool in patient evaluation. Its use is, however, limited because of the invasive nature and the required expertise in sample evaluation.

**Table 3:** Suggested laboratory tests in serum or urine for screening of secondary factors

General, for indication	
Serum	
Calcium and phosphate homeostasis	Calcium (corrected for albumin), phosphate, 25OHD
Chronic kidney disease	Creatinine
Bone turnover	ALP (with GGT or bone-specific) and BTMs if avalable (CTX, PINP)
Hematologic diseases	Blood cell count
Inflammation	ESR or CRP
Diabetes mellitus (type 1 or 2)	Fasting glucose and HbA1C
Hyperthyroidism	TSH
Hypogonadism, male	Testosterone
Urine	
Hypercalciuria	24 h urine calcium and creatinine
On indication based on:	
Abnormal serum calcium or phosphate	PTH
Decreased TSH	FT4
Suspicion mastocytosis	Tryptase
Suspicion hemochromatosis	Ferritin
Hypophosphatasia	ALP (decreased level)
Decreased testosterone	LH, FSH, SHBG, prolactine
Amenorrhea	Estradiol, FSH
Suspicion acromegaly	IGF1
Suspicion Cushing’s syndrome	Urinary cortisol, dexamethasone suppression, midnight salivary cortisol
Suspicion malabsorption, celiac disease	Anti-TTG, antibodies against endomysium, fat soluble vitamins

*25OHD* 25-hydroxyvitamin D, *ALP* alkaline phosphatase, *GGT* γ-glutamyltransferase, *BTMs* bone turnover markers, *CTX* serum carboxy-terminal cross-linking telopeptide of type I collagen, *PINP* serum procollagen type I N propeptide, *ESR* erythrocyte sedimentation rate, *CRP* C-reactive protein, *TSH* thyroid-stimulating hormone, *PTH* parathyroid hormone, *LH* luteinizing hormone, *FSH* follicle-stimulating hormone, *SHBG* sex hormone-binding globulin, *IGF1* Insulin-like growth factor 1, *anti-TTG* antibodies to tissue transglutaminase

### Approaches to Genetic Testing

A thorough clinical examination of a patient with early-onset osteoporosis may give some indications regarding the underlying genetic cause, for example blue sclerae, joint laxity and dentinogenesis imperfecta in OI or bony protrusions in the skull or a history of transient facial nerve palsy in *SGMS2*-related osteoporosis. However, clinical presentations vary and in most of the monogenic forms, no specific clinical signs have been reported. Family history, both regarding the patient’s parents and siblings, but also the patient’s own children, may give helpful clues and aide in establishing the genetic nature of the disease. In most situations, however, a genetic evaluation is in any case necessary to confidently exclude or confirm a heritable disease.

As mentioned, approximately 90% of all patients with OI have mutations in *COL1A1* or *COL1A2*, the two genes encoding type I collagen. These should be included in genetic screening since the presentation of OI can be variable and in many instances the patients lack the typical clinical appearance of OI. Several other monogenic forms of bone fragility have been recognized and it is therefore advisable to use one of the several commercially available, or in-house, gene panels for OI and monogenic osteoporosis. Another possibility is to perform exome sequencing or whole genome sequencing and filter the sequencing data to detect variants of known clinical importance in the selected genes. This approach would allow the re-analysis of the sequencing data as novel disease-causing genes are identified.

At least patients with a positive family history for early-onset osteoporosis would benefit from a thorough genetic investigation, as identification of the defective gene can help to establish long-term prognosis, enable genetic counseling, and may influence treatment decisions. Since possibilities for genetic studies vary in different centers, guidelines for approaches and prioritizing need to be established locally. Recent developments in testing capacity and prices are promising and with decreasing costs, genetic testing can be more widely implemented in our clinical practices.

## Treatment Considerations in Early-Onset Osteoporosis

Because osteoporosis is rare in the young, only few large-scale studies with pharmacological treatment have been performed and most of these studies had BMD and not fractures as a primary outcome. Concerning supplementation with calcium and vitamin D, studies with fracture outcome are lacking but an increase in BMD has been observed in some smaller scale studies [102, 103]. In case of insufficiency, also of other vitamins and minerals, supplementation is a pragmatic approach as well as other lifestyle advises related to use of alcohol, smoking and exercise. In fact, in children and adolescents with recurrent fractures, studies have indicated low calcium intake, vitamin D deficiency and inadequate physical activity to be a major contributing factor [15] and these should be addressed before other medications are considered.

Treatment of underlying diseases or secondary factors appears to be beneficial for bone and BMD increases have been observed e.g., with diet in celiac disease, (9% increase of radius BMD after one year), anti-TNF in IBD, estrogens for amenorrhea, surgery for primary hyperparathyroidism and Cushing’s disease, treatment of hyperthyroidism and malnutrition [19]. When treatment of the underlying cause is not possible or effective and fracture risk appears high, antiresorptive and anabolic drugs can be considered, taking into account potential adverse effects in pregnancies in women of childbearing age. The use of zoledronic acid in premenopausal breast cancer patients has clearly shown in the Austrian Breast and Colorectal cancer study group 12 trial (ABCSG-12) to prevent adjuvant endocrine therapy related bone loss [104] but evidence for fracture prevention is limited. A management algorithm for early breast cancer patients including premenopausal women on adjuvant endocrine therapy was recently published [23]. Treatment with bisphosphonates has also shown to improve BMD in several other underlying conditions of osteoporosis in young people such as anorexia nervosa (mainly at the lumbar spine), IBD, cystic fibrosis, thalassemia major and glucocorticoid-induced bone loss but fracture data are mostly lacking [14]. For guidance on treatment of glucocorticoid-induced osteoporosis in the young we refer to guidelines of the IOF-ECTS in 2012 and de American College of Rheumatology in 2017 [31, 105]. In PLAO, a retrospective, multicenter study in 52 women showed that BMD increased without pharmacological treatment but more so during treatment with bisphosphonates and with even higher increase in BMD with teriparatide although in all three groups about 19% developed a new fracture during follow-up of 36 months [106]. A similar larger increase in LS BMD was reported in a retrospective study of 32 PLAO women with multiple fractures treated with teriparatide for one year (15.5% ± 6.6) compared to controls (7.5% ± 7.1) [107].

Awaiting further RCTs with fracture reduction as a primary outcome in young persons with osteoporosis, a personalized approach is needed depending on the patient and the condition, where an absence of evidence should not be equal to evidence of absence of effects. The management of children and young adults with osteoporosis and fragility fractures requires a patient-centered multidisciplinary approach with a team of health professionals, optimally with expertise in both pediatric and young adult bone disease.

Apart from OI, even less data from human treatment trials is available for monogenic forms of osteoporosis. These treatment-related aspects have been briefly discussed in the separate paragraphs dealing with each genetic form. In young women with known osteoporosis and fragility fractures that desire future pregnancy it is important to discuss timing of pregnancy regarding the effect of pregnancy and especially of lactation on their bone health and the timing of bone-active medication. In a recent small-scale case–control study no major teratogenic effects of bisphosphonates were observed but potential negative effect on rates of neonatal complications and live birth rate could not be excluded [108]. Because of retention of bisphosphonates in bone it is generally advised not to start bisphosphonate treatment if when there are plans for future pregnancy within 1 year [109]. There is no data in humans on the safety of teriparatide, denosumab or romosozumab in pregnant women but since these drugs are not retained in bone it can be assumed that after stopping them before a pregnancy, they will not have teratogenic effects. What is not known is whether their effects will remain if no after-treatment with bisphosphonates is given, nor is it known if there is the same risk of stopping denosumab as in postmenopausal women on a rebound of bone turnover and occurrence of multiple vertebral fractures [110].

## Concluding Remarks

Early-onset osteoporosis continues to be a diagnostic challenge. Careful clinical, radiological and biochemical evaluation is needed to detect underlying secondary causes. These should always precede a suspicion of a monogenic form of osteoporosis. Further, when establishing a diagnosis of idiopathic osteoporosis, a careful genetic evaluation is also needed to exclude the known monogenic forms of osteoporosis. Recent developments in advanced radiological imaging techniques that can measure volumetric BMD and bone microstructure in cancellous and cortical bone and estimate bone strength, such as high-resolution pQCT, may in the future give more insight into underlying bone defects and may limit the need for invasive bone biopsies. Because there is very limited evidence of anti-fracture efficacy of bone-active drugs it is important to consider their use in a personalized approach, after implementing optimal lifestyle and calcium and vitamin D supplementation and treatment of the underlying disorder while considering plans for future pregnancy in females of child-bearing age.

In the genetic forms of early-onset osteoporosis, careful characterization of the associated phenotypes, tissue-level pathology and the involved cellular mechanisms are of great value. Such studies can lead to discoveries that will benefit not only patients with these particular rare disorders but may prove efficacious even in the treatment of other patients with early-onset osteoporosis or patients with postmenopausal osteoporosis. Genetic diagnosis provides the affected individuals and their families information about the cause of osteoporosis and the mode of inheritance. The results will also affect the patients' medical care and follow-up. A specific genetic diagnosis enables early detection and timely preventive measures also in other family members who are affected by the same genetic defect. A multidisciplinary approach with a team of experts including e.g., pediatric and adult internists and endocrinologists, orthopedic surgeons, obstetricians and geneticists is usually needed for optimal care of these young patients with osteoporosis and fragility fractures. In order to expand the knowledge on the rare forms of osteoporosis in children and young adults, international collaboration is important, as is increasingly being implemented for example in the European Networks for Rare Bone Conditions (ERN BOND) and rare endocrine disorders (ERN ENDO) and within scientific consortia like GEFOS and GENOMOS and the GEMSTONE COST action (http://www.gefos.org; http://www.genomos.eu; https://cost-gemstone.eu).
